# Palliative care national plan implementation through stakeholder analysis

**DOI:** 10.1186/s12904-024-01427-1

**Published:** 2024-07-01

**Authors:** Miguel Antonio Sánchez-Cárdenas, Marta Ximena León-Delgado, Lina María Vargas-Escobar, Sofia Elizabeth Muñoz Medina, Paula Milena Buitrago Florian, David Andrade Fonseca, Juan Esteban Correa-Morales

**Affiliations:** 1 Faculty of Nursing, El Bosque University, Bogotá, DC Colombia; 2 Palliative care program, La Sabana University, Bogotá, DC Colombia

**Keywords:** Palliative care, Stakeholder analysis, National plan, Public health, Colombia

## Abstract

**Background:**

National palliative care plans depend upon stakeholder engagement to succeed. Assessing the capability, interest, and knowledge of stakeholders is a crucial step in the implementation of public health initiatives, as recommended by the World Health Organisation. However, utilising stakeholder analysis is a strategy underused in public palliative care.

**Objective:**

To conduct a stakeholder analysis characterising a diverse group of stakeholders involved in implementing a national palliative care plan in three rural regions of an upper-middle-income country.

**Methods:**

A descriptive cross-sectional study design, complemented by a quantitative stakeholder analysis approach, was executed through a survey designed to gauge stakeholders’ levels of interest and capability in relation to five fundamental dimensions of public palliative care: provision of services, accessibility of essential medicines, palliative care education, financial support, and palliative care vitality. Stakeholders were categorised as promoters (high-power, high-interest), latent (high-power, low-interest), advocates (low-power, high-interest), and indifferent (low-power and low-interest). Stakeholder self-perceived category and knowledge level were also assessed.

**Results:**

Among the 65 surveyed stakeholders, 19 were categorised as promoters, 34 as advocates, 9 as latent, and 3 as indifferent. Stakeholders’ self-perception of their category did not align with the results of the quantitative analysis. When evaluated by region and palliative care dimensions the distribution of stakeholders was nonuniform. Palliative care funding was the dimension with the highest number of stakeholders categorised as indifferent, and the lowest percentage of promoters. Stakeholders categorised as promoters consistently reported a low level of knowledge, regardless of the dimension, region, or their level of interest.

**Conclusions:**

Assessing the capability, interest, and knowledge of stakeholders is a crucial step when implementing public health initiatives in palliative care. It allows for a data-driven decision-making process on how to delegate responsibilities, administer financial resources, and establish governance boards that remain engaged and work efficiently.

**Supplementary Information:**

The online version contains supplementary material available at 10.1186/s12904-024-01427-1.

## Introduction

The imperative for worldwide palliative care, as a fundamental health intervention that effectively mitigates health-related suffering, has prompted a universal call to action spanning all nations [1]. The 2020 Berlin Declaration, endorsed by the three leading international palliative organisations, emphasises the necessity for the concrete integration of public health palliative care initiatives into national health strategies, as well as their incorporation into regional and global health frameworks [2]. In 2021, the World Health Organisation released a set of adaptable indicators tailored to countries’ income levels. These indicators aim to enhance comprehension, facilitate inequity mitigation, and offer strategic recommendations for the development of countries’ public health palliative care [3]. According to the most recent Palliative Care Atlas, 55 out of 198 countries have a national strategy or plan for palliative care, with varying levels of implementation [4]. The most comprehensive evaluation of national palliative care plans released in 2023, utilising WHO indicators as a framework for analysis, concluded that the effective execution of these plans hinges on five factors: adequate funding, a dedicated coordinating institution, regular monitoring, and the active engagement of all relevant stakeholders [5]. The empirical experience, as seen in countries such as Ireland, which was the first country to implement a national palliative care plan, validates the significance of these factors in achieving their policy analysis goals [6]. Likewise, countries at the forefront of palliative care integration into healthcare systems, such as Canada, have underscored the importance of a large-scale coordination approach that encompasses and engages all relevant stakeholders [7]. This call to action, along with the aforementioned challenges, has predominantly been embraced by high-income countries in the global north, while nations with middle and low incomes in the global south, including Africa, Asia, Latin America, and the Mediterranean region, are lagging [5, 9].

According to the World Bank classification [8], Colombia is an upper-middle-income Latin American country with a generalised palliative care provision [9]. In the last seven years, Colombia has become a pioneer in palliative public health development, despite being a war and drug-torn country. Standing as one of the limited number of countries hosting a National Palliative Care Observatory, this institution has made it possible to gather public health data to track the evolution of palliative care progress within the country. Their endeavours culminated in the first analysis of geographical disparities, achieved by applying international indicators in a national context [10]. Nonetheless, as highlighted by Marshall et al., merely recognising the imbalanced development of palliative care falls short in the absence of a rural, tightly coordinated, and multidisciplinary model that seamlessly integrates across local healthcare environments, through information systems and care planning [11]. Therefore, the Colombian National Observatory of Palliative Care (OCCP) embarked on a two-stage task. The first stage was to identify barriers to accessing palliative care through a social mapping approach that engaged various stakeholders, concentrating on rural regions marked by greater geographical disparities [12]. The second stage was to build up a national plan to tackle palliative care inequality through multi-stakeholder platforms. This national plan excels due to its financial solvency and the novelty of its methodology, described in a phase-by-phase manner in a prior publication, to exemplify and make feasible its replication by other countries [13]. Currently, the OCCP, serving as the coordinating body responsible for executing the Colombian palliative care plan, has directed its efforts towards prioritising the three most underserved rural regions, where access to palliative care services is alarmingly low, at less than 0.1 services per 100,000 inhabitants. The challenge is to create alliances among stakeholders, based on their willingness, competence, and capability, to ensure a collaborative effort in executing the national palliative care plan [5, 11, 14]. We conducted a comprehensive survey to explore and analyse stakeholders’ commitment. We hypothesise that by prospectively assessing the engagement and capabilities of local stakeholders, data-driven decisions can be made to assign responsibilities, thereby increasing the chances of successfully implementing the Colombian palliative care public health strategy.

### Methodology

A cross-sectional study design coupled with a quantitative stakeholder analysis was chosen to describe the stakeholders’ commitment to the national palliative care plan. A stakeholder is an entity, whether an individual or an organisation, with a vested interest in the promotion of a policy. Stakeholder analysis involves systematically gathering and analysing information to identify whose interests align with policy or programme development and/or implementation. Identifying key actors and assessing their knowledge, interests, positions, alliances, and significance concerning a policy enables more effective interactions, greater support for the policy, addressing potential misunderstandings or opposition, and enhancing the chances of policy or program success [15]. The World Bank has outlined how to map the stakeholder’s analysis result in a power-interest grid, divided into four profiles: promoters (high-power, high-interest stakeholders who want to collaborate and remain fully engaged), latent (high-power, low-interest stakeholders who can have a lot of influence over the project but do not want to be involved in the details), advocate (low-power, high-interest stakeholders who can offer great insights and ideas for the project but with limited resources), and indifferent parties(stakeholders with low-power and low-interest) [16]. To classify each stakeholder within one of the four groups, we conducted a survey covering 5 dimensions of palliative care development embedded in the palliative care plan: provision of services of palliative care, availability of essential medicines for palliative care, palliative care education, palliative care funding, and palliative care vitality, understood as the level of people and community empowerment [3, 17]. A detailed explanation of how the stakeholders’ responses were quantitatively calculated within each specific power-interest group is further provided in the data analysis section. Based on the Franco-Trigo et al. systematic scoping review [18], we followed the RISA tool to ensure adequate stakeholder analysis reporting quality and the STROBE guidelines on cross-sectional studies to ascertain high-quality reporting [19].

### Participants and recruitment

Invited stakeholders had to be individuals or institutions working as local health authorities, health insurance companies, healthcare institutions, independent professionals, or universities with nursing and medicine faculties. These stakeholders were located in three Colombian regions (Amazonas, Orinoquía, and Pacífico), which were identified as having no known palliative care activity or capacity-building palliative care activity, according to the international classification in palliative care development [10]. Invited stakeholders could also refer other potential participants. No time criteria regarding experience were required. The recruitment process was performed through different means. A public announcement was made through the Colombian Health Ministry, the OCCP website, local government authorities, and various social media channels. Personalised invitations through email were also sent to stakeholders who had participated in the construction of the national palliative care plan. Stakeholders who had previously declined to participate in the development of the national palliative care plan or had previous interactions with the OCCP were also contacted. As the study was exploratory, we opted not to perform a formal sample estimation but rather focused on recruiting as many participants as possible.

### Data collection

An electronic survey link was included in the invitation email. Additionally, two follow-up emails were sent at four-week intervals to promote survey participation, with no incentives or compensation offered for taking part. The survey was conducted using SurveyMonkey, and received content approval from all involved researchers. Each dimension consisted of a varying number of questions. A pilot survey was conducted with 13 participants, including researchers not involved in the survey design and peers knowledgeable in palliative care and public health. The purpose of the test was to evaluate the comprehensibility of the survey and the time required for completion. Following the pilot test, a feedback session was held to carefully assess and clarify any ambiguous concepts. As a result of the pilot test, explanations of each dimension were added for respondent convenience. The survey commenced with an introductory paragraph and an electronic informed consent form, which all participating stakeholders were needed to complete. In addition, demographic information was collected on stakeholders’ locations, affiliations, and professional profiles. Stakeholders also self-assessed their stakeholder type as promoters, latent, advocates, or indifferent. On a scale from 0 to 100 (with 100 the highest score) stakeholders rated both their level of knowledge of local palliative care and their overall willingness to implement the Colombian palliative care plan. Finally, stakeholders shared their input by answering questions related to the palliative care dimensions that they considered best aligned with their professional profiles. The survey consisted of 66 questions, categorised as follows: 14 on the provision of palliative care services, 8 on the availability of essential medicines for palliative care, 16 on palliative care education, 4 on palliative care funding, and 24 on palliative care vitality. The full survey can be found in supplementary material S1.

### Data analysis

Each question of the survey evaluated the stakeholder’s capability and interest using a five-point Likert-type scale ranging from 1 (lowest) to 5 (highest). To conduct the stakeholder analysis and establish each stakeholder profile, the sum of the stakeholder responses was calculated for each dimension, distinguishing the results for both capability and interest. Collected data were transferred to an anonymized Excel sheet for subsequent analysis. For quantitative variables, measures of central tendency (mean, median) and measures of dispersion such as standard deviation were calculated. Likewise, position measures such as percentiles and interquartile ranges were calculated for the responses of each dimension. Similarly, for each of the dimensions, according to the results of the Likert scale, the results were dichotomised based on the median obtained. A median score of 4 or 5 was considered a high level of engagement, while a median score of 3 or less was considered a low level of engagement. Meanwhile, for qualitative variables, absolute and relative frequencies were calculated. The data were analysed using the statistical software R version 4.3.1.

## Results

A total of 163 professionals were invited through mail from March 16 to July 15 of 2023, eliciting 65 responses, resulting in a 39,8% response rate. The participating stakeholders were nonuniformly distributed, 36 were from the Amazonas region(55.38%), 18 were from the Pacífico region (27.69%), and 11 were from the Orinoquía region (16.92%). The majority of participants were healthcare institutions and independent health professionals, followed by local health authorities. Universities and administrative entities had few or no representatives in some regions. Moreover, in all three regions, the majority of stakeholders identified themselves as advocates of palliative care, with very few considering themselves indifferent to palliative care. Among the 65 surveyed stakeholders, 19 were categorised as promoters, 34 as advocates, 9 as latent, and 3 as indifferent. In terms of the stakeholders’ knowledge and disposition, assessed on a scale from 0 to 100, the disposition component had a higher result than the knowledge component in the three regions. In the Pacífico region, there was a notable disparity between the two components, whereas in the other two regions, the scores were relatively similar. Overall, the mean disposition score was 61,9(± 35,4), and the mean knowledge score was 52,2 (± 28,2) for the three regions. Table 1 shows the detailed data on the stakeholder affiliation, self-perception, knowledge, and disposition components by region.

**Table 1:** Characteristics of the stakeholders in the three regions of Colombia

Results	Amazonas N: 36 n (%)	Orinoquía N: 11 n (%)	Pacífico N: 18 n (%)	Total N: 65 n (%)
**Type of institution**				
Local health authorities	13 (36,1)	2 (18, 1)	6 (33,3)	21 (32,3)
Administrative entities	1 (2,7)	0 (0)	4 (22,2)	5 (7,6)
Health-providing institutions and independent professionals	22 (61,1)	7 (63,6)	6 (33,3)	35 (53,8)
Universities with programs in Medicine and Nursing	0 (0)	2 (18,1)	2 (11,1)	4 (6,1)
**Position**				
Promoter	9 (25)	2 (18,1)	8 (44,4)	19 (29,2)
Advocate	20 (55,5)	7 (63,6)	8 (44,4)	35 (53,8)
Latent	6 (16,6)	1 (9,0)	2 (11,1)	9 (13,8)
Indifferent	1 (2,7)	1 (9,0)	0 (0)	2 (3,0)
**Knowledge and disposition**				
Interest, mean (SD)	56,4 (± 35,9)	56,9 (± 38,6)	76,0 (± 30)	61.9 (± 35,4)
Knowledge, mean (SD)	49,3 (± 27,7)	54,6 (± 29,9)	56,6 (± 29,3)	52.2 (± 28,2)

SD: standard deviation

### Stakeholder analysis per public health dimension

**Fig. 1:**
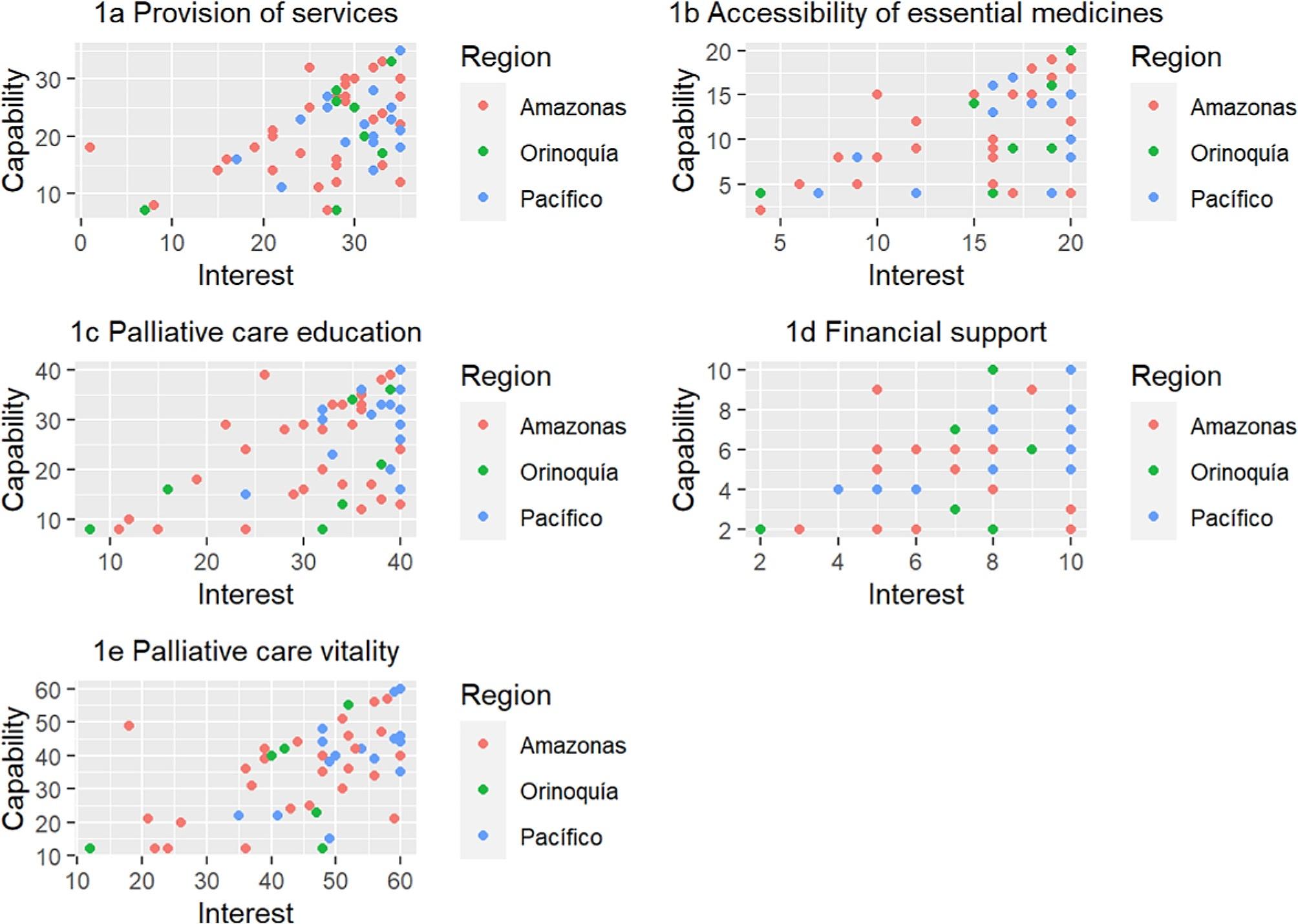
**a**. Stakeholders’ capability and interest in provision of services; **b**. Stakeholders’ capability and interest for accessibility in essential medicines; **c**. Stakeholders’ capability and interest in palliative care education; **d**. Stakeholders’ capability and interest in financial support; **e**. Stakeholders’ capability and interest in palliative care vitality

For the public health dimension concerning the provision of palliative care services, stakeholders were classified as follows: 44.6% as promoters, 32.3% as advocates, 21.5% as part of the crowd, and 1.5% as latent. In the dimension of ensuring the availability of essential medicines for palliative care, stakeholders were categorised as follows: 40.6% as advocates, 37.5% as promoters, 20.3% as part of the indifferent, and 1.5% as latent. Regarding palliative care education: 53,8% of stakeholders were categorised as promoters, 29,2% as advocates, 13,8% indifferent, and 3% as latent. In the palliative care funding dimension, 38,4% of stakeholders were categorised as promoters, 36,9% as indifferent, and 24,6% as advocates. No latent stakeholders were identified for this dimension. Finally, stakeholders in the palliative care vitality dimension were distributed as 42,6% promoters, 31,1% advocates, 24,5% indifferent, and 1,6% as latent. Figure 1a, b, c and d, and 1e show the stakeholders’ capability and interest per region and public palliative care dimension.

### Stakeholder analysis per region

**Fig. 2:**
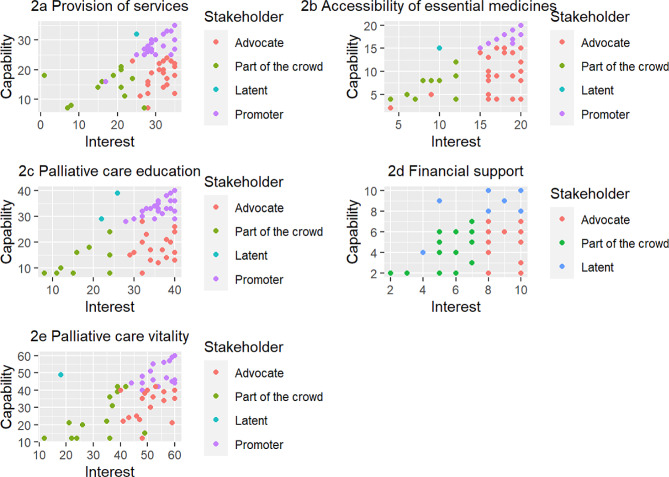
**a**. Stakeholder analysis for provision of services; **b**. Stakeholder analysis for accessibility of essential medicines; **c**.Stakeholder analysis for palliative care education; **d**. Stakeholder analysis for financial support; **e**. Stakeholder analysis for palliative care vitality

Although the regions share a similar level of palliative care development, it is worth noting that only in the Amazonas region did a small percentage (3%) of stakeholders qualify as latent across the dimensions of provision of palliative care services, availability of essential medicines, palliative care education, and palliative care vitality, excluding palliative care funding. In the other two regions, no stakeholders were identified as latent. The Amazonas region appears to have a sizeable percentage (42–47%) of stakeholders who are promoters of the dimensions of palliative care services and palliative care education. The stakeholder analysis also shows that the Pacífico region has a considerable percentage (45–67%) of stakeholders who are promoters for the dimensions of palliative care services, availability of essential medicines, and palliative care vitality. Likewise, the Orinoquía region has a significant percentage (45–55%) of stakeholders who are promoters for the dimensions of palliative care services, availability of essential medicines, and palliative care education. Palliative care funding was the dimension within the three regions, with the highest percentage (22–44%) of stakeholders categorised as indifferent, and the lowest percentage (36–39%) of stakeholders categorised as promoters. Figure 2a, b, c and d, and 2e depict in more detail the distribution of the stakeholder analysis distributed in the public palliative care dimensions.

### Stakeholders’ knowledge and disposition

**Fig. 3:**
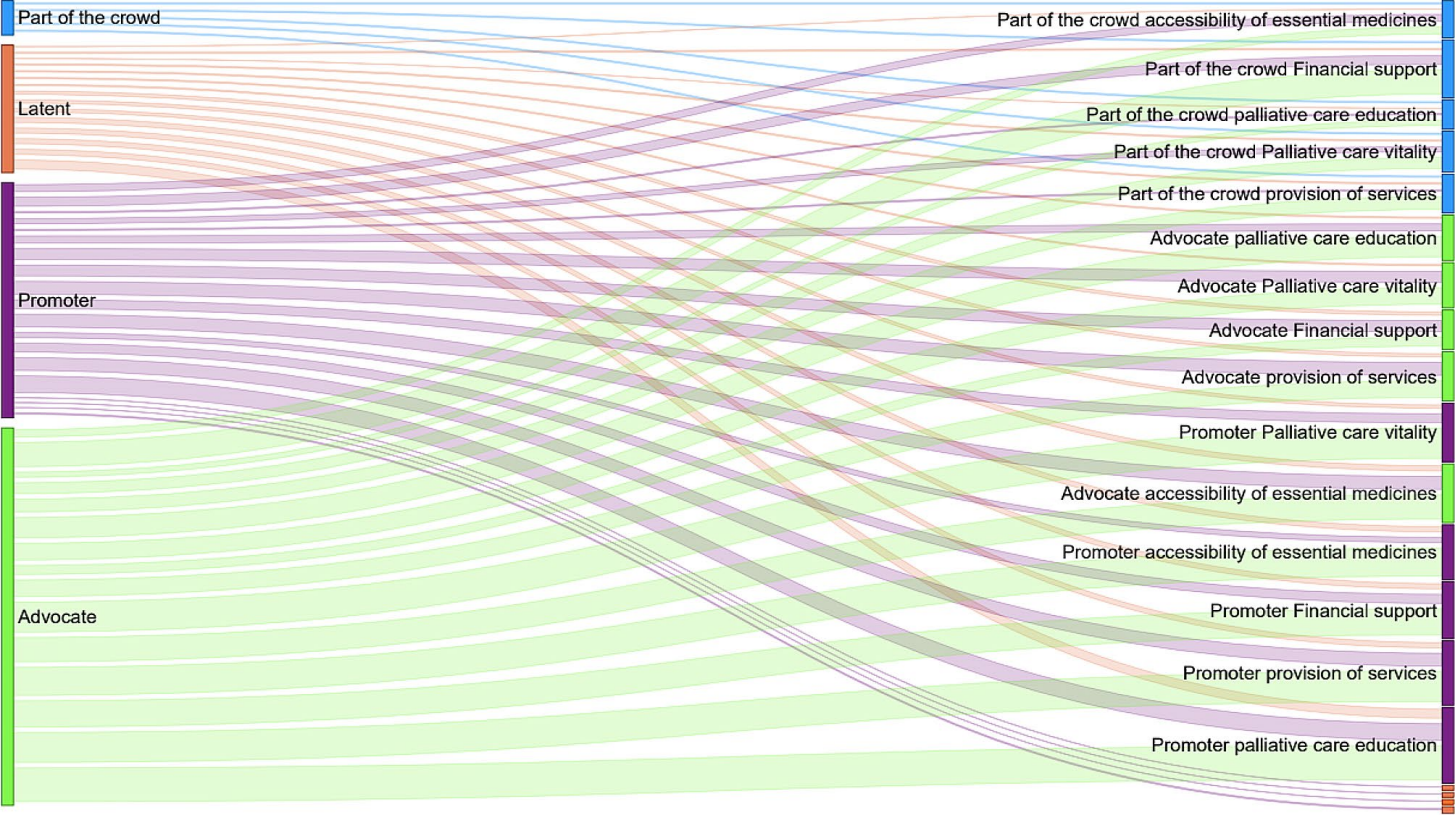
Stakeholders’ self perceived categorisation vs. stakeholder analysis result

In all three regions, stakeholders reported a stronger disposition than knowledge. The Kruskal-Wallis hypothesis test revealed no significant differences in the levels of interest or knowledge among these regions (*p* < 0.05). When examining the data for each dimension of public health palliative care, a noteworthy finding emerged. A substantial percentage of stakeholders classified as promoters rated their knowledge as 50 or lower on a scale of 1 to 100. Specifically, in the dimension related to palliative care services, this applied to 42% of stakeholders in the Amazonas region, 60% in the Orinoquía region, and 57% in the Pacífico region. Similarly, for the dimension concerning the availability of essential medicines for palliative care, 53% of promoters in the Amazonas region, 50% in the Orinoquía region, and 80% in the Pacífico region reported a low level of knowledge. In the realm of palliative care education, 35% of promoters in the Amazonas region, 60% in the Orinoquía region, and 50% in the Pacífico region acknowledged having limited knowledge. For the dimension of palliative care funding, 42% of promoters in the Amazonas region, 33% in the Orinoquía region, and 50% in the Pacífico region indicated a relatively low level of knowledge. In the palliative care vitality dimension, 25% of promoters in the Amazonas region, 33% in the Orinoquía region, and 55% in the Pacífico region reported a lower grade of knowledge. For more detailed information on the levels of knowledge and interest for each type of stakeholder and dimension, please refer to supplementary material S2. Ultimately, Fig. 3 illustrates the comparison between stakeholders’ self-perceived categorisation at the beginning of the survey and the categorisation results obtained through the quantitative analysis conducted in the stakeholder analysis.

## Discussion

Our cross-sectional stakeholder-based survey plays a vital role in supporting the Colombian public health plan for palliative care, which is in effect until 2026. A stakeholder analysis was conducted to optimise resource allocation, improve efficiency, and facilitate more effective delegation of responsibilities to local stakeholders, all with the aim of maximising results. The results indicate that within the three least developed regions in Colombia regarding palliative care, stakeholders are distributed as follows: 29.2% are promoters (high-power, high-interest), 53.8% are advocates (low-power, high-interest), 13.8% are latent stakeholders (high-power, low-interest), and 3% are indifferent stakeholders (low-power, low-interest). Mapping these profiles aids decision-makers in being knowledgeable when consulting, engaging, and involving stakeholders [20]. Our survey illustrated remarkable insights into stakeholder characteristics. For example, stakeholders did not exhibit the same level of engagement across all the palliative care dimensions, irrespective of their prior categorisation. We observed that stakeholders displayed a greater level of engagement with regard to the dimensions of service provision and palliative care education, whereas they showed indifference toward the financial dimension. Additionally, we noted differences in the stakeholder categorisation among the three Colombian regions. For instance, the Pacífico region boasts the highest percentage of promoters in the domains of palliative care education and vitality. Meanwhile, the Orinoquía region exhibits the highest percentage of promoters in service provision and palliative care medications. In contrast, the Amazonas region stands out as the only region where stakeholders were categorised as latent. The differences pertaining to the stakeholders’ dimensions of commitment and regional contrasts lay the challenge of implementing the national palliative care plan and the importance of having conducted this stakeholder analysis. As the role that a large-scale coordinator is called to play, balancing and overviewing the local stakeholders’ implementing efforts.

Furthermore, the results indicate that there is a discrepancy between the stakeholders’ self perceived category and the quantity categorisation we performed. This lack of congruency indicates that stakeholders may underestimate or overestimate their engagement level, and thus position themselves incorrectly. While stakeholder analysis is typically conducted using qualitative methods, this finding underlies the benefit of the quantitative methodology we employed as an iterative approach with broad possibilities of data analysis [21, 22]. Finally, our results reveal that the level of knowledge a stakeholder may have, is not necessarily correlated with their level of interest. Recognising that local stakeholders who are promoters require continuous education in palliative care as they implement the national palliative care plan is a major finding that highlights the dynamic nature of public health strategies, where the key actors have ongoing needs to be able to excel.

A substantial body of evidence supports the significance of researching ways to enhance stakeholder engagement as a crucial factor for the successful implementation of effective health-related interventions [23–25]. However, many of the public health strategies in palliative care are not backed by a stakeholder analysis [5]. We are aware of only one previous national plan that adopted this approach in its implementation process. The Uganda palliative care plan for 2017–2021 established four main dimensions (capacity development, advocacy, research, and governance/resource mobilisation). Stakeholders, including the Ministry of Health, palliative care association members, service providers, and donors, were categorised by their influence and interest levels [26]. The extent to which this categorisation contributed to the successful implementation of the Uganda palliative care plan remains unknown. We consider it inappropriate to compare their stakeholder analysis to ours due to differences in methodology, dimensions considered, and the types of stakeholders involved. Nonetheless, it is foreseeable that this strategy will have a significant impact on the implementation of the national plan, as it addresses the failures observed in previous experiences of forming collaboration networks in other countries [6, 7]. Moreover, since the latest mapping of palliative care national plans worldwide [5], additional low-middle-income nations such as Jordan [27], Malawi [28], Benin [29], and Saudi Arabia [30], among others, have established or begun building their palliative care national plans. These countries may benefit from our methodology to characterize their stakeholders and advance the implementation of their own palliative care plans. We believe the methodology used herein could also be adapted to other health or public contexts and improved with the integration of artificial intelligence [31].

Our study has several limitations to take into consideration. We had a low rate of responses to our survey, which may limit the interpretation of our results. Especially in the Oriniquía region, where only 11 stakeholders answered the survey. Additionally, the results reported a small or nonexistent percentage of stakeholders categorised as latent. Although this finding may be legitimate, there is a possibility that the way the quantitative analysis was conducted resulted in the underrepresentation of this category. The novel quantitative methodology used to determine the stakeholder’s level of capability and interest in each dimension is certainly improvable by a mixed methodology. Using a 0-100 scale to assess the level of stakeholders´ knowledge is based on self perception and is not a validated instrument, but it gives an idea of the stakeholders´ needs. The type of stakeholders surveyed could have been expanded to have a wider representation of the local societies in each region. Last, we followed the RISA tool to ensure adequate stakeholder analysis reporting quality, however, this tool is not specific to palliative care and requires some grade of adaptation to be fully followed through. Additional research is needed to evaluate the influence of stakeholder analysis on the implementation of public health palliative care initiatives and to determine the most effective methodology for conducting stakeholder analysis in palliative care.

## Conclusions

Assessing the capability, interest, and knowledge of stakeholders is a crucial step in public health initiatives. It facilitates a decision-making process guided by data, enabling the allocation of responsibilities, management of financial resources, and formation of engaged and efficient governance boards. We used this strategy, enhanced by a quantitative analysis, for the initial phase of implementing a national palliative care plan for an upper-middle-income country that aims to transform three rural regions in each of the public health dimensions proposed by the World Health Organization for palliative care development. We believe this pilot and successful attempt may be replicated and improved by other countries advancing in the integration of public health palliative care.

### Electronic supplementary material

Below is the link to the electronic supplementary material.

Supplementary Material 1

## Data Availability

Data on the national palliative care plan will be. Available as an official document in the website of the Colombian Observatory for Palliative Care.
