# Supplementary material for: Palliative care national plan implementation through stakeholder analysis
Source: BMC Palliat Care. 2024 Jul 1;23:163. doi: 10.1186/s12904-024-01427-1 (PMC11218343; doi:10.1186/s12904-024-01427-1)

**Supplementary material S1:**

The complete survey can be found here in spanish: <https://es.surveymonkey.com/r/ZB3SDJF>. The following is a translated version for the readers' convenience:

PARTICIPANT'S DECLARATION:

I declare that I am acting consciously, freely, and voluntarily as a participant in this research, contributing to the information collection phase, and that I accept the information detailed here.

If you agree to the terms stipulated here, you may proceed with the proposed virtual activity.

1.Do you accept the informed consent and agree to take the survey? yes/no

2.What is your name?

**3. In which department of the country do you reside or work the majority of your time?**

**4. Institution you represent:**

- Local health authorities

- Health Benefit Plan Administrators (EAPB - Health insurers)

- Health care providers and independent professionals

- Media

- Universities with Medicine and Nursing programs

- Non-governmental organization

5. Position:

6. Your level of knowledge about palliative care in your region is: 0(low) - 100 (high)

7. Your position regarding a strategy for building palliative care networks is:

- Promoter (actors actively working to improve access and quality of palliative care)

- Advocate (actors considering palliative care as a fundamental tool to improve health and quality of life)

- Indifferent (actors having little or no interest in building palliative care networks)

- Latent (actors not directly involved in building palliative care networks, but can influence implementation and outcomes)

8. Your willingness to actively lead the creation of a palliative care network is: 1 (low) - 5 (high)

SERVICE DELIVERY OF PALLIATIVE CARE

Please consider the definitions of interest and capability.

Definition of capability: It is the ability of the interested party to influence the implementation of Palliative Care. Consider the relationship between the amount of resources and the ability to mobilize them.

Definition of interest: Disposition and inclination that the interested party has in the project.

Please select the option according to the following instruction, 1 being the lowest interest and capability, and 5 being the highest interest and capability.

9. Increasing the supply of specialized palliative care services:

Interest 1(low)- 5 (high)

Capability 1(low)- 5 (high)

10. Generating alliances with indigenous health systems to implement palliative care mechanisms in these communities.

Interest 1(low)- 5 (high)

Capability 1(low)- 5 (high)

11. Generating interdepartmental alliances to increase specialized access to palliative care

Interest 1(low)- 5 (high)

Capability 1(low)- 5 (high)

12. Increasing the capacity of home care programs to offer quality palliative care

Interest 1(low)- 5 (high)

Capability 1(low)- 5 (high)

13. Increasing support from benefit plan administrators to providers at different levels of care to ensure continuity of palliative care, especially during care transitions.

Interest 1(low)- 5 (high)

Capability 1(low)- 5 (high)

14. Implementing a telemedicine program in palliative care to guide patient management in regions without palliative care services

Interest 1(low)- 5 (high)

Capability 1(low)- 5 (high)

15. Creating technical-scientific consultations for the development and implementation of minimum standards for enabling palliative care services.

Interest 1(low)- 5 (high)

Capability 1(low)- 5 (high)

ESSENTIAL MEDICATIONS

Please consider the definitions of interest and capability.

Definition of capability: It is the ability of the interested party to influence the implementation of Palliative Care. Consider the relationship between the amount of resources and the ability to mobilize them.

Definition of interest: Disposition and inclination that the interested party has in the project.

Please select the option according to the following instruction, 1 being the lowest interest and capability, and 5 being the highest interest and capability.

16. Establishing a monitoring program for the use and distribution of opioid medications in the territory, considering the needs, availability, and accessibility of special control medications.

Interest 1(low)- 5 (high)

Capability 1(low)- 5 (high)

17. Increase the number of licensed pharmacies with 24/7 availability for dispensing controlled medications.

Interest 1(low)- 5 (high)

Capability 1(low)- 5 (high)

18. Increase electronic prescription of opioids to improve monitoring of these medications.

Interest 1(low)- 5 (high)

Capability 1(low)- 5 (high)

19. Increase research on the rational and appropriate use of opioids in children with palliative needs.

Interest 1(low)- 5 (high)

Capability 1(low)- 5 (high)

PALLIATIVE CARE EDUCATION

In this space, we seek to understand the availability of undergraduate, postgraduate, and continuing education resources and/or research projects in palliative care in the regions.

Please select the option according to the following instruction, 1 being the lowest interest and capability, and 5 being the highest interest and capability.

20. Promote training of health and community personnel in pediatric palliative care.

Interest 1(low)- 5 (high)

Capability 1(low)- 5 (high)

21. Offer continuing education programs in palliative care for primary health care professionals.

Interest 1(low)- 5 (high)

Capability 1(low)- 5 (high)

22. Develop a training program aimed at stakeholders involved in the formulation and dispensing of controlled medications.

Interest 1(low)- 5 (high)

Capability 1(low)- 5 (high)

23. Conduct research on rational opioid use.

Interest 1(low)- 5 (high)

Capability 1(low)- 5 (high)

24. Incorporate teaching of palliative care into health sciences programs through specific subjects, cross-cutting contents, or elective subjects.

Interest 1(low)- 5 (high)

Capability 1(low)- 5 (high)

25. Design a mentoring program with palliative care specialists to guide and accompany the development of new palliative care programs and chairs in health sciences programs of higher education.

Interest 1(low)- 5 (high)

Capability 1(low)- 5 (high)

26. Implement education programs mediated by information and communication technologies to train health personnel in various parts of the national territory.

Interest 1(low)- 5 (high)

Capability 1(low)- 5 (high)

27. Include palliative care training in technical health programs, health volunteers, and spiritual advisors.

Interest 1(low)- 5 (high)

Capability 1(low)- 5 (high)

FINANCING

In this space, we aim to understand the availability of resources to ensure funding for palliative care as part of the health benefits plan.

Interest 1(low)- 5 (high)

Capability 1(low)- 5 (high)

Please select the option according to the following instruction, 1 being the lowest interest and capability, and 5 being the highest interest and capability.

Interest 1(low)- 5 (high)

Capability 1(low)- 5 (high)

28. Manage relationships with benefit plan administrators to ensure funding for palliative care services.

Interest 1(low)- 5 (high)

Capability 1(low)- 5 (high)

29. Create a communication plan aimed at benefit plan administrators showing the economic benefits, quality of care, and user and family satisfaction derived from palliative care.

Interest 1(low)- 5 (high)

Capability 1(low)- 5 (high)

VITALITY

In this section, you will find a series of statements for which we ask you to consider the definition of interest and capability.

Definition of capability: It is the ability of the interested party to influence the implementation of Palliative Care. Consider the relationship between the amount of resources and the ability to mobilize them.

Definition of interest: Disposition and inclination that the interested party has in the project.

In this domain, we will analyze the region's capacity to empower individuals, families, and communities as partners in the development of social and health services, participation in decision-making, availability of promotional resources to improve the participation of patients and caregivers in the development of palliative care programs.

Please select the option according to the following instruction, 1 being the lowest interest and capability, and 5 being the highest interest and capability.

30. Implement a regional program of support and social assistance to individuals with palliative care needs and their families

Interest 1(low)- 5 (high)

Capability 1(low)- 5 (high)

31. Develop a regional volunteer program linking individuals interested in providing support to individuals with palliative care needs and their families in both hospital and community settings

Interest 1(low)- 5 (high)

Capability 1(low)- 5 (high)

32. Promote the creation of support networks for individuals with palliative care needs and their families, particularly in highly cohesive social groups

Interest 1(low)- 5 (high)

Capability 1(low)- 5 (high)

33. Foster strategic partnerships with municipal influencer groups to train volunteers

Interest 1(low)- 5 (high)

Capability 1(low)- 5 (high)

34. Create coordinated actions with existing patient associations

Interest 1(low)- 5 (high)

Capability 1(low)- 5 (high)

35. Conduct a mapping of social assets in health to activate the volunteer network

Interest 1(low)- 5 (high)

Capability 1(low)- 5 (high)

36. Prioritize caregiver support in chronic disease programs

Interest 1(low)- 5 (high)

Capability 1(low)- 5 (high)

37. Consolidate collaborative working groups between healthcare professionals and media for the creation of an understanding culture around advanced illness and the role of palliative care throughout life

Interest 1(low)- 5 (high)

Capability 1(low)- 5 (high)

38. Use local media to disseminate palliative care content in the general community

Interest 1(low)- 5 (high)

Capability 1(low)- 5 (high)

39. Strengthen communication of palliative care associations and teams through strategic alliances with academia and communication programs

Interest 1(low)- 5 (high)

Capability 1(low)- 5 (high)

40. Consolidate workshops with journalists for positive communication around palliative care from academia and scientific associations

Interest 1(low)- 5 (high)

Capability 1(low)- 5 (high)

41. Develop lobbying actions with health authorities to promote palliative care in your region

Interest 1(low)- 5 (high)

Capability 1(low)- 5 (high)

We appreciate your responses; these will be shared and disseminated later.

**Supplementary material S2**
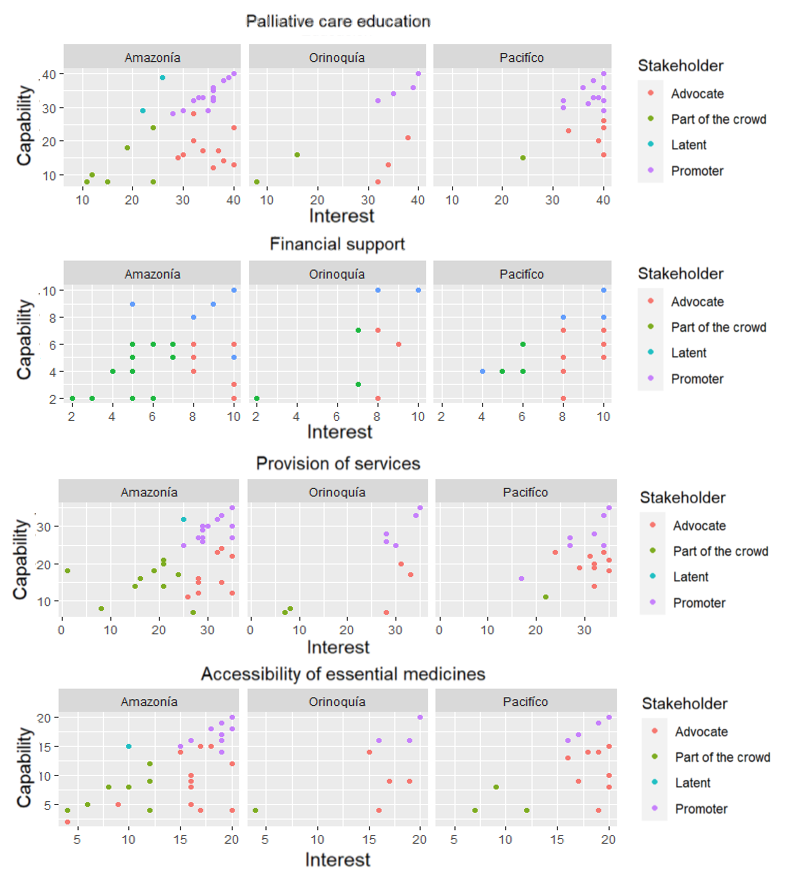


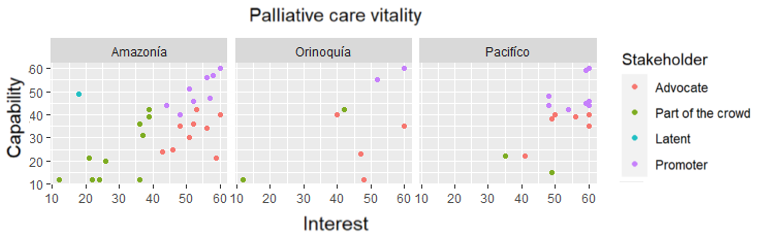

Supplement: Supplementary file 1 — Supplementary Material 1 [file 12904_2024_1427_MOESM1_ESM.docx]
